# Dose dependent effect of statins on postoperative atrial fibrillation after cardiac surgery among patients treated with beta blockers

**DOI:** 10.1186/1749-8090-4-61

**Published:** 2009-11-04

**Authors:** Salima Mithani, Muhammad S Akbar, Deborah J Johnson, Michael Kuskowski, Katherine K Apple, Jana Bonawitz-Conlin, Herbert B Ward, Rosemary F Kelly, Edward O McFalls, Hanna E Bloomfield, Jian-Ming Li, Selcuk Adabag

**Affiliations:** 1 Department of Internal Medicine, Veterans Affairs Medical Center, Veterans Drive, Minneapolis 55417, USA; 2 Department of Medicine, University of Minnesota, Delaware St SE, Minneapolis 55455, USA; 3 Division of Cardiology, Veterans Affairs Medical Center, Veterans Drive, Minneapolis 55417, USA; 4 Division of Cardiology, University of Minnesota, Delaware St SE, Minneapolis 55455, USA; 5 Division of Cardiovascular Surgery, Veterans Affairs Medical Center, Veterans Drive, Minneapolis 55417, USA; 6 Geriatric Research Education Center, Veterans Affairs Medical Center, Veterans Drive, Minneapolis 55417, USA; 7 Center for Chronic Disease Outcomes Research, Veterans Affairs Medical Center, Veterans Drive, Minneapolis 55417, USA

## Abstract

**Background:**

Previous studies on the effects of Statins in preventing atrial fibrillation (AF) after cardiac surgery have shown conflicting results. Whether statins prevent AF in patients treated with postoperative beta blockers and whether the statin-effect is dose related are unknown.

**Methods:**

We retrospectively studied 1936 consecutive patients who underwent coronary artery bypass graft (CABG) (n = 1493) or valve surgery (n = 443) at the Minneapolis Veterans Affairs Medical Center. All patients were in sinus rhythm before the surgery. Postoperative beta blockers were administered routinely (92% within 24 hours postoperatively).

**Results:**

Mean age was 66+10 years and 68% of the patients were taking Statins. Postoperative AF occurred in 588 (30%) patients and led to longer length of stay in the intensive care unit versus those without AF (5.1+7.6 days versus 2.5+2.3 days, p < 0.0001). Patients with a past history of AF had a 5 times higher risk of postoperative AF (odds ratio 5.1; 95% confidence interval 3.4 to 7.7; p < 0.0001). AF occurred in 31% of patients taking statins versus 29% of the others (p = 0.49). In multivariable analysis, statins were not associated with AF (odds ratio (OR) 0.93, 95% confidence interval (CI) 0.7 to 1.2; p = 0.59). However, in a subgroup analysis, the patients treated with Simvastatin >20 mg daily had a 36% reduction in the risk of postoperative AF (OR 0.64, 95% CI 0.43 to 0.6; p = 0.03) in comparison to those taking lower dosages.

**Conclusion:**

Among cardiac surgery patients treated with postoperative beta blockers Statin treatment reduces the incidence of postoperative AF when used at higher dosages

## Background

Postoperative atrial fibrillation (AF) occurs after 30-40% of cardiac surgeries [1-3] and is associated with increased risk of stroke [2-5], longer hospitalization, higher cost [4-6] and greater risk of long-term mortality [7]. Beta blockers [8-12] and amiodarone [13-15] are known to reduce the incidence of postoperative AF after cardiac surgery but the effects of statins have been less conclusive [16]. While statin treatment appeared to lower the risk of postoperative AF in some initial observational studies [17-21] no benefit was noted in a recent, well-conducted cohort of >4000 patients [22]. In the only randomized clinical trial in this arena, Atorvastatin, started 7 days before cardiac surgery, was associated with a > 60% reduction in the incidence of postoperative AF among 200 patients undergoing coronary artery bypass graft (CABG) surgery [23]. However, the extraordinarily high AF rate (~60%) in the control group of this study was not representative of the experience at most centers [3,17-21]. Furthermore, beta blockers, which unequivocally reduce postoperative AF, were not administered routinely after surgery and the number of patients undergoing concomitant valve surgery was small (n = 41). Whether statin treatment prevents AF among patients receiving postoperative beta blockers is still unknown. Also, whether the statin effect on postoperative AF is dosage dependent is unclear. Thus, the aim of the present investigation was to fill these gaps in knowledge in a large cohort of patients undergoing CABG or valve surgery.

## Methods

### Study population

This study was approved by the human studies subcommittee of the Research and Development Committee of the Minneapolis Veterans Affairs (VA) Medical Center. Individual consent was waived. A total of 2207 patients underwent CABG or valve surgery (with or without concomitant CABG) at the Minneapolis VA Medical Center between February 1999 and November 2005. Of these, 271 patients were excluded because of permanent preoperative AF (n = 131) or missing/uninterpretable electrocardiograms (ECG). A total of 1,936 patients were included in the final analysis, including those with a previous history of AF who were in sinus rhythm at the time of surgery (n = 114).

### Data Collection

Preoperative clinical variables, procedural details and laboratory test results were retrospectively abstracted from the patients' electronic medical records and the VA Continuous Improvement in Cardiac Surgery Program, which is an ongoing database of prospectively-collected data in all patients undergoing heart surgery within the VA system [24-27]. Pre and postoperative medications, including statins and beta blockers, were obtained from the VA pharmacy database and further confirmed by the clinician notes in the electronic medical records. The use of VA pharmacy refill data as a measure of actual medication use has previously been validated [28,29]. At our institution all preoperative medications are continued postoperatively and all patients receive beta blockers within 24 hours after surgery, unless contraindicated. The peri-operative ECGs were obtained from the ECG laboratory database at the Minneapolis VA Medical Center.

### Ascertainment of atrial fibrillation

The primary outcome variable was development of AF within 30 days after the cardiac surgery. Postoperatively, cardiac rhythm was continuously monitored for the first 72 to 96 hours in the intensive care and the telemetry step-down units. Subsequently, 12-lead ECGs were performed routinely on a daily basis and when clinically-indicated until patients were discharged from the hospital. A follow-up ECG was performed at 30-days after hospital discharge. All ECGs were interpreted by two physicians (ASM and MSA). Additional revisions were performed by a cardiologist (ASA) when necessary.

### Data analysis

All statistical analyses were performed using SPSS (version 16). Distribution of variables in the study cohorts were summarized as mean + one standard deviation when normally distributed or median and interquartile range if skewed. We compared baseline characteristics of the statin treated and untreated patients, using *t *test for continuous variables and likelihood ratio *X*^2 ^tests for categorical variables. Logistic regression analysis was used to determine the odds ratio (OR) of AF in association with statin treatment. Multivariable logistic regression models included the baseline differences between the statin and non-statin groups and predictors known to be associated with postoperative AF. Thus the predictor variables in our multivariable models were age, body mass index, prior history of AF, chronic lung disease, diabetes mellitus, hypertension, left ventricular function, peripheral and cerebral vascular disease, smoking status, history of myocardial infarction, New York Heart Association functional class, beta blocker treatment status, calcium channel blocker treatment, digoxin treatment, cardiomegaly, surgical procedure, cross-clamp time and statin treatment status. As a separate analysis a propensity score for taking statin was created for each patient using the variables that were different between the statin-treated and untreated groups. Multivariable models including the propensity score were created to assess the statin effect on AF.

Predetermined subgroup analyses were performed to assess statin effect in patients undergoing CABG surgery only and to investigate whether the AF incidence was related to the dosage of Statin. A p-value of < 0.05 was considered statistically significant.

## Results

### Patient Characteristics

The baseline characteristics of the 1936 cardiac surgery patients in relation to preoperative statin treatment are outlined in Table 1. Mean age was 66+10 years and risk factors known to be associated with postoperative AF were prevalent (Table 1). A total of 1322 (68%) patients were treated with statins pre- and postoperatively. Of these, 1205 were taking Simvastatin (mean dosage 33+22 mg; range 5 to 80 mg). The patients receiving statin treatment were younger, had a higher prevalence of coronary risk factors and were more likely to undergo CABG in comparison to those not taking statins (Table 1). On the other hand, statin-untreated patients were more likely to have left ventricular dysfunction, cardiomegaly and symptoms of heart failure.

**Table 1:** Baseline characteristics of the 1,936 cardiac surgery patients included in the study

		**Statin Treatment**		
				
	**All patients** **n = 1936**	**Yes** **n = 1322**	**No** **n = 614**	**p value**
**Clinical data**				
Age [years]	66+10	65+10	67+10	0.01
Male, %	99%	99%	99%	0.46
BMI [kg/m^2^]	29.4+5.4	30+5.4	29+5.3	0.03
Diabetes mellitus, %	34%	35%	30%	0.03
Hypertension, %	60%	81%	73%	0.001
Chronic lung disease, %	23%	22%	26%	0.06
Peripheral vascular disease, %	30%	30%	30%	0.90
Cerebrovascular disease, %	22%	22%	21%	0.49
Current Smoking, %	68%	21%	17%	0.03
History of myocardial infarction, %	42%	46%	33%	<0.0001
History of heart surgery, %	9%	9%	9%	0.82
LV ejection fraction <50%, %	41%	42%	47%	0.05
NYHA heart failure class III/IV, %	35%	33%	39%	0.01
History of atrial fibrillation, %	5.7%	6.3%	4.6%	0.13
Chronic kidney disease, %	3.7%	11%	13%	0.53
Cardiomegaly, %	27%	25%	31%	0.01
**Preoperative medications**				
BetaBlockers, %	53%	57%	46%	<0.0001
ACE-I or ARB, %	72%	73%	69%	0.59
Gemfibrozil, %	8%	8%	7%	0.59
Calcium Chanel blocker, %	21%	23%	17%	0.01
Amiodarone, %	16%	16%	17%	0.31
Diuretics, %	41%	40%	43%	0.13
Digoxin, %	8%	7%	12%	<0.0001

Continuous variables are expressed as mean + SD or median with interquartile range

Abbreviations: LV: left ventricular; NYHA:New York Heart Association; ACE-I: Angiotensin Converting Enzyme Inhibitor; ARB: Angiotensin Receptor Blocker;BMI: Body Mass Index

Of the 1936 surgical procedures, 1493 (77%) were CABG only (Table 2). Postoperatively, 1778 (92%) of the patients received beta-blockers within the 1^st ^24 hours after surgery.

**Table 2:** Details of the surgical procedures performed on the 1,936 study patients

		**Statin Treatment**		
			
	**All patients** **n = 1936**	**Yes** **n = 1322**	**No** **n = 614**	**p value**
CABG, %	77%	84%	62%	<0.0001
Valve surgery, %	12%	7%	23%	<0.0001
Combined CABG and valve surgery, %	9%	8%	12%	<0.0001
Other surgical procedure, %	2%	1%	3%	<0.0001
Off-pump surgery, %	5%	5%	5%	0.94
Urgent/emergent surgery, %	11%	12%	10%	0.20
Ischemic time [minutes]	92+40	90+38	95+42	0.04
Total CPB time [minutes]	137+51	134+50	140+53	0.08

Abbreviations: CABG: coronary artery bypass surgery; CBP: Cardiopulmonary bypass

### Postoperative AF

A total of 588 (30%) patients developed postoperative AF after a median 2 days (range 1 to 29 days) following cardiac surgery. Postoperative beta blocker use (p = 0.01), cardiomegaly (p = 0.01), and previous history of AF (p = 0.001) were associated with AF. Patients with a previous history of AF had a 5 times higher incidence of postoperative AF (odds ratio 5.1; 95% confidence interval 3.4 to 7.7; p < 0.0001) compared to those without a prior history. Patients who developed postoperative AF had a significantly longer length of stay in the Intensive Care Unit (5.1+7.6 days versus 2.5+2.3 days, p < 0.0001) than those who maintained sinus rhythm.

### Effect of Statins

Postoperative AF occurred in 31% of statin-treated patients versus 29% of those not taking statins (p = 0.49). In multivariable analysis, after adjusting for the differences between the statin and non-statin groups and for predictors known to be associated with postoperative AF (full list under statistical methods), statin treatment was not associated with the risk of postoperative AF (odds ratio 0.93, 95% confidence interval 0.7 to 1.2; p = 0.59). Results were similar after performing propensity matching analysis, to adjust for baseline differences between the statin-treated and untreated groups. Results did not change when the analysis was limited to patients who underwent CABG only.

### Effect of Statin dosage

The majority (91%) of our statin-treated patients were taking Simvastatin. Of these 1205 patients treated with Simvastatin, 668 (55%) were taking Simvastatin <20 mg daily versus 537 treated with a higher dosage. Notably, postoperative AF was less common among the patients taking a higher dosage of statins versus those taking <20 mg/day (28% vs. 34%; p = 0.03). In multivariate analysis, the patients treated with statins>20 mg daily had a 36% reduction in the risk of postoperative AF (odds ratio 0.64, 95% confidence interval 0.43 to 0.6; p = 0.03) in comparison to those taking Simvastatin <20 mg daily (results similar with propensity matching).

## Discussion

The aim of this investigation was to assess whether statins prevented AF after CABG and/or valve surgery among patients who were treated with beta blockers in the immediate postoperative period. We found that postoperative AF (~30% in our cohort) occurred more commonly in those with a previous history of AF and was associated with a longer length of stay in the Intensive Care Unit. There was a 36% reduction in postoperative AF among those who were treated with a higher dosage (i.e. > 20 mg/day) of statins. However the incidence of postoperative AF was not influenced by low dose statin treatment.

There has been a recent interest in using statins for preventing postoperative AF after cardiac surgery, however, the clinical results are mixed. Initial cohort studies in this arena suggested that statin treatment was associated with a 40% to 50% reduction in the incidence of postoperative AF in patients undergoing CABG [17-21]. Further, in a small randomized clinical trial of 200 statin-naïve patients, Atorvastatin 40 mg, started 7 days prior to elective cardiac surgery, was associated with a > 60% reduction in the risk of postoperative AF in comparison to placebo [23]. However, there were some limitations in these studies. First, postoperative beta blockers were not routinely administered and their use was not uniformly reported. Second, only a few valve surgery patients were included. Third, the AF rate in the placebo arm of the only randomized clinical trial was extraordinarily high (57%). Finally, the magnitude of benefit approaching 60% is considered unusually high with present medical treatments. The positive findings in these studies were recently refuted by a well-conducted cohort study of >4000 consecutive patients undergoing CABG or valve surgery [22]. In this study, Virani et al. found that statin treatment did not influence the incidence of postoperative AF.

Our data shows that, when beta blockers are on board, higher doses of statins were required to reduce the incidence of postoperative AF. Previously, Lertsburapa et al [17] and Kourliouros et al [18] had also found that higher dose statins were associated with greater reduction in postoperative AF. While our statin-treated patients were predominantly taking Simvastatin, a combination of different statins were used in the other 2 studies suggesting that the statin-effect is not brand specific.

The most notable difference between the previous studies and the present investigation was the use of postoperative beta blockers. Indeed, >90% of our patients were treated with beta blockers within 24 hours of cardiac surgery. Administering beta blockers after surgery reduce AF incidence and withdrawal of preoperative beta blockers is one of the strongest predictors of postoperative AF [8-12]. Thus, it is possible that the previous reports of substantial benefits with statins may have been confounded by suboptimal utilization of postoperative beta blockers, particularly in the early postoperative period.

Another notable difference between the current and past study cohorts was in the number of valve surgery patients included. Postoperative AF is more common after valve surgery. Further, in contrast to patients undergoing CABG, statins are not routinely clinically indicated in valve surgery patients. Thus, assessment of statin effect on postoperative AF is particularly important among these patients. Whereas, most previous studies were largely comprised of patients undergoing CABG, almost 25% of our cohort and >30% of the study cohort by Virani et al. underwent valve surgery with or without concomitant CABG. We found no evidence of reduction in postoperative AF with statins in the subgroup of our cohort who had valve surgery.

There is suggestive evidence that statin treatment may reduce non-surgical AF among patients with coronary heart disease [30,31]. However, the mechanism remains unknown and randomized clinical trial data are lacking. Notably, in a large cohort study in which propensity scoring was utilized to adjust for the baseline differences between the statin and non-statin groups there was no effect on AF incidence [32].

One of the strengths of our investigation was the size of the study sample of nearly 2000 patients, of which ~30% developed postoperative AF. This large sample afforded us a greater statistical power to adjust for all of the measured differences between the statin vs. no-statin groups and the predictor variables that are known to be associated with postoperative AF. Routine use of postoperative beta blockers and inclusion of a substantial number of valve surgery patients are other notable strengths. On the other hand, this study also has some limitations. The inherent short-comings of retrospective cohort study design, including baseline differences among study groups, cannot be completely avoided, by a large sample size and statistical adjustment for the multiple variables. Further, the findings in subgroup analyses, although pre-specified in this case, should be considered as hypothesis generating. Almost all of our study patients were male. Thus caution should be exercised in extending these results to women. Also, the duration of statin treatment was not known. It is possible that the effect of statins on AF is dependent upon the duration of statin-therapy.

## Conclusion

In conclusion, in this large cohort of cardiac surgery patients who were routinely treated with postoperative beta blockers, 30% had postoperative AF associated with a longer stay at the intensive care unit. Higher dose (but not lower dose) statin treatment was associated with a 36% reduction in the risk of postoperative AF.

## Competing interests

The authors declare that they have no competing interests.

## Authors' contributions

ASM: Study design, data acquisition, analysis and interpretation of data, drafting and revision of manuscript and final approval. MSA: Study design, data collection, revision and final approval of manuscript. DJJ: Study design, data collection, revision and final approval of manuscript. MK: Data analysis and interpretation, revision and final approval of manuscript. KKA: Data collection, revision and final approval of manuscript. JBC: Data collection, revision and final approval of manuscript. HBW: Study design, interpretation of data, revision and final approval of manuscript. RFK: Study design, interpretation of data, revision and final approval of manuscript. EOM: Study design, interpretation of data, revision and final approval of manuscript. HEB: Study design, interpretation of data, revision and final approval of manuscript. JML: Study design, interpretation of data, revision and final approval of manuscript. ASA: Conception and study design, data acquisition, analysis and interpretation of data, drafting and revision of manuscript and final approval.

## References

[B1] Echahidi N, Pibarot P, O'Hara G, Mathieu P (2008). Mechanisms, prevention and treatment of atrial fibrillation after cardiac surgery. J Am Coll Cardiol.

[B2] Creswell LL, Schuessler RB, Rosenbloom M, Cox JL (1993). Hazards of postoperative atrial arrhythmias. Ann Thorac Surg.

[B3] Andrews TC, Reimold SC, Berlin JA, Antman EM (1991). Prevention of supraventricular arrhythmias after coronary artery bypass surgery: A meta-analysis of randomized control trials. Circulation.

[B4] Hogue CW, Creswell LL, Gutterman DD, Fleisher LA (2005). Epidemiology, mechanisms, and risks: American College of Chest Physicians guidelines for the prevention and management of postoperative atrial fibrillation after cardiac surgery. Chest.

[B5] Mathew JP, Parks R, Savino JS, Friedman AS, Koch C (1996). Atrial fibrillation following coronary artery bypass graft surgery: predictors, outcomes, and resource utilization. MultiCenter Study of Perioperative Ischemia Research Group. JAMA.

[B6] Aranki SF, Shaw DP, Adams DH, Rizzo RJ, Couper GS (1996). Predictors of atrial fibrillation after coronary artery surgery. Current trends and impact on hospital resources. Circulation.

[B7] Villareal RP, Hariharan R, Liu BC, Kar B, Lee VV (2004). Postoperative atrial fibrillation and mortality after coronary artery bypass surgery. J Am Coll Cardiol.

[B8] Bradley D, Creswell LL, Hogue CW, Epstein AE, Prystowsky EN, Daoud EG (2005). American college of chest physicians guidelines for the prevention and management of postoperative atrial fibrillation after cardiac surgery. Chest.

[B9] Mathew JP, Fontes ML, Tudor IC, Ramsay J, Duke P (2004). A multicenter risk index for atrial fibrillation after cardiac surgery. JAMA.

[B10] Coleman CI, Perkerson KA, Gillespie EL, Kluger J, Gallagher R (2004). Impact of prophylactic post-operative beta-blockade on post-cardiothoracic surgery length of stay and atrial fibrillation. Ann Pharmacother.

[B11] Connolly SJ, Cybulsky I, Lamy A, Roberts RS, O'brien B (2003). Double-blind, placebo-controlled, randomized trial of prophylactic metoprolol for reduction of hospital length of stay after heart surgery: the beta-Blocker Length Of Stay [BLOS] study. Am Heart J.

[B12] Ferguson TB, Coombs LP, Peterson ED (2002). Preoperative beta-blocker use and mortality and morbidity following CABG surgery in North America. JAMA.

[B13] Mitchell LB, Exner DV, Wyse DG, Connolly CJ, Prystai GD (2005). Prophylactic Oral Amiodarone for the Prevention of Arrhythmias that Begin Early After Revascularization, Valve Replacement, or Repair: PAPABEAR: a randomized controlled trial. JAMA.

[B14] Guarnieri T, Nolan S, Gottlieb SO, Dudek A, Lowry DR (1999). Intravenous amiodarone for the prevention of atrial fibrillation after open heart surgery: the Amiodarone Reduction in Coronary Heart [ARCH] trial. J Am Coll Cardiol.

[B15] Daoud EG, Strickberger SA, Man KC, Goyal R, Deeb GM (1997). Preoperative amiodarone as prophylaxis against atrial fibrillation after heart surgery. N Engl J Med.

[B16] Howard PA, Barnes BJ (2008). Potential use of statins to prevent atrial fibrillation after coronary artery bypass surgery. Ann Pharmacother.

[B17] Lertsburapa K, White CM, Kluger J, Faheem O, Hammond J, Coleman CI (2008). Preoperative statins for the prevention of atrial fibrillation after cardiothoracic surgery. J Thorac Cardiovasc Surg.

[B18] Kourliouros A, De Souza A, Roberts N, Marciniak A, Tsiouris A (2008). Dose- related effects of statins on atrial fibrillation after cardiac surgery. Ann Thorac Surg.

[B19] Ozaydin M, Dogan A, Varol E, Kapan S, Tuzon N (2007). Statin use before by-pass surgery decreases the incidence and shortens the duration of postoperative atrial fibrillation. Cardiology.

[B20] Mariscalco G, Lorusso R, Klersy C, Ferrarese S, Tozzi M (2007). Observational study on the beneficial effect of preoperative statins in reducing atrial fibrillation after coronary surgery. Ann Thorac Surg.

[B21] Marín F, Pascual DA, Roldán V, Arribas JM, Ahumada M (2006). Statins and postoperative risk of atrial fibrillation following coronary artery bypass grafting. Am J Cardiol.

[B22] Virani S, Nambi V, Razavi M, Lee W, Elayda M (2008). Preoperative statin therapy is not associated with a decrease in the incidence of postoperative atrial fibrillation in patients undergoing cardiac surgery. Am Heart J.

[B23] Patti G, Chello M, Candura D, Pasceri V, D'Ambrosio A (2006). Randomized trial of atorvastatin for reduction of postoperative atrial fibrillation in patients undergoing cardiac surgery: results of the ARMYDA-3 [Atorvastatin for Reduction of MYocardial Dysrhythmia After cardiac surgery] study. Circulation.

[B24] Adabag AS, Rector T, Mithani S, Harmala J, Ward HB (2007). Prognostic significance of elevated cardiac troponin I after heart surgery. Ann Thorac Surg.

[B25] Grover FL, Johnson RR, Shroyer AL, Marshall G, Hammermeister KE (1994). The Veterans Affairs Continuous Improvement in Cardiac Surgery Study. Ann Thorac Surg.

[B26] Grover FL, Johnson RR, Marshall G, Hammermeister KE (1993). Factors predictive of operative mortality among coronary artery bypass subsets. Ann Thorac Surg.

[B27] Grover FL, Hammermeister KE, Burchfiel C (1990). Initial report of the Veterans Administration Preoperative Risk Assessment Study for Cardiac Surgery. Ann Thorac Surg.

[B28] Osterberg L, Blaschke T (2005). Adherence to medication. N Engl J Med.

[B29] Steiner JF, Koepsell TD, Fihn SD, Inui TS (1988). A general method of compliance assessment using centralized pharmacy records: description and validation. Med Care.

[B30] Goldberger JJ, Subacius H, Schaechter A, Howard A, Berger R (2006). Effects of statin therapy on arrhythmic events and survival in patients with nonischemic dilated cardiomyopathy. J Am Coll Cardiol.

[B31] Mitchell LB, Powell JL, Gillis AM, Kehl V, Hallstrom AP, AVID Investigators (2003). Are lipid-lowering drugs also antiarrhythmic drugs? An analysis of the Antiarrhythmic Versus Implantable Defibrillators [AVID] Trial. J Am Coll Cardiol.

[B32] Adabag S, Nelson D, Bloomfield H (2007). Effects of statin therapy on preventing atrial fibrillation in coronary disease and heart failure. Am Heart J.

